# Evaluation of residual monomer release after polymerization of different restorative materials used in pediatric dentistry

**DOI:** 10.1186/s12903-022-02260-9

**Published:** 2022-06-13

**Authors:** Gülsüm Duruk, Sibel Akküç, Yılmaz Uğur

**Affiliations:** 1 Department of Pediatric Dentistry, Faculty of Dentistry, Inonu University, Malatya, Turkey; 2 Department of Pharmacy Services, Health Services Vocational School, Inonu University, Malatya, Turkey

**Keywords:** Dental material, Compomer, BIS-GMA; HEMA, TEGDMA, UDMA, HPLC–PDA

## Abstract

**Background:**

The choice of the restorative resin material to be used in pediatric dentistry is of a great importance due to the cytotoxic effects caused by residual monomers. In this study, it was aimed to investigate the amount of residual monomer released over time from different resin-based restorative materials, which are widely used in pediatric dentistry, by using high performance liquid chromatography with photodiode array detector (HPLC–PDA).

**Methods:**

The compomers in all colors (Twinky Star and Glasiositte A_2_), two composites with different hybrid properties (Arabesk-GrandioSO), and RMGIC (Ionolux) samples with 2 × 5 mm diameters were prepared. The samples were polymerized with an LED light unit (CELALUX 2, VOCO, Cuxhaven, Germany) and then finishing-polishing procedures were applied. A total of 156 samples were obtained, 13 samples in each of the 12 groups. The amount of residual monomer (BIS-GMA; HEMA, TEGDMA, UDMA) (µg/mL) released into the 75% ethanol solution was determined at different times, (1st hour, 1st, 7th, 14th, and 21st day) by using HPLC–PDA.

**Results:**

The residual monomer release continued on day 21 and BIS-GMA was the most released monomer in all groups. HEMA release showed a maximum increase in all the materials at day 7. The highest amount of residual monomer was detected in the gold-colored compomer. HEMA and BIS-GMA release from RMGIC was less than others in all time frames.

**Conclusions:**

The color and composition of resin-based restorative materials affect the amount of residual monomer. Pediatric dentists should prefer gold-colored compomers less than others as a restorative material, especially in deep cavities. More studies are needed about the subject.

## Significance statement

This study will change the perspectives of pediatric dentists and manufacturers on the resin-based restorative materials and shed light on the restorative material choices of pediatric dentists and future studies.

## Introduction

The use of the light-cured resin based materials such as composite, compomer (polyacid-modified composite resin), resin modified glass ionomer cement (RMGIC) developed as an alternative to amalgam in pediatric dentistry has become increasingly widespread [1]. The aesthetics of the composites, the fluoride release of the RMGIC and compomers, and different color options, which is more acceptable in children, make these materials preferable [2–4].

Long chain and high molecular weight compounds formed through the bonds of resin monomers in aesthetic restorative materials and adhesive systems with chemical bonds are called 'polymers', and this reaction, which occurs with the reaction of carbon double bonds between monomers, is called 'polymerization' [5]. However, due to the inability of all carbon double bonds to react, some unreacted monomers may remain in their position, which is called 'residual monomer' [6].

The amount of residual monomer in light-cured restorative materials depends on many factors such as the composition of the resin material, the amount and the concentration of the solvent used, the type of light-curing unit (LCU), light intensity, power and duration, room temperature, presence of oxygen, humidity, the thickness of the resin layer, and the amount and type of pigment [3, 4].

Monomers such as hydroxy ethyl methacrylate (HEMA), bisphenol-A glycidyl methacrylate (BIS-GMA), triethylene glycol dimethacrylate (TEGDMA), urethane dimethacrylate (UDMA) form the organic matrix part of the light-cured restorative materials [7, 8]. Gentoxic, mutagenic, and allergic effects of these residual monomers, which are released into saliva as a result of polymerization that does not take place in full efficiency, are well-known [9–12], and it has been stated that Bisphenol-A (BPA), a component of BIS-GMA, increases estrogenic activities [13], TEGDMA causes apoptosis of gingival fibroblasts [14, 15], and HEMA has cytotoxic effects in dentin and pulp [16].

Çelik et al. [1] evaluated the cytotoxic effects of the compomer (Dyract XP) and microhybrid resin based composite (Filtek Z 250) dental restorative materials on human gingival fibroblast cells and reported that the tested materials in three different periods caused significant decreases in the cell viability rates compared to the control group. The cell viability was observed for compomer as 56%, 44%, and 73% in freshly prepared samples, at seven-day aging, and at 21-day aging, respectively. These rates were 40%, 33%, and 35% in composite resin samples.

The choice of restorative resin material to be used in pediatric dentistry is of a great importance due to the harmful effects of residual monomers. To date, most of the studies on residual monomer released have deal with dental composite resins [7, 15, 17–24]. However, less information is available about the compomers [8, 25], especially colored compomers [26]. As far as we know, there is no study examining the amount of residual monomer release from composites, RMGIC, and all the colors of the colored compomers in the color scale within the same study protocol. Therefore, this study aims to shed light on the selection of restorative materials in pediatric dentisrtry by investigating the amount of residual monomer released from different colored compomers, composites with different hybrid properties, and RMGIC after polymerization using the HPLC–PDA method.

The null hypothesis of this study was that the color/shade and hybrid features of the resin-based restorative materials do not cause any difference in the amount of monomer release.

## Methods

### Determination of study groups

Two different hybrid composites, 9 different colored packable compomers, and one RMGIC were tested. The study was conducted for twelve different groups: Groups 1 to 8: Packable Compomer (Twinky Star, VOCO, Germany)- Group 1: Berry; Group 2: Lemon; Group 3: Orange; Group 4: Green; Group 5: Gold; Group 6: Pink; Group 7: Blue; Group 8: Silver- Group 9: White–Shade A_2_ Packable Compomer (Glasiosite, VOCO, Germany); Group 10: RMGIC (Light-Curing Glass Ionomer Cement–Ionolux, VOCO, Germany); Group 11: Shade A_2_ Light-Curing Micro-Hybrid Composite–Arabesk; Group 12: Shade A_2_ Universal Nano-Hybrid Composite (GrandioSO, VOCO, Germany). The materials and their compositions, manufacturers, and lot numbers are listed in Table 1.

**Table 1:** The monomer contents of the resin restorative materials, included in the company's safety data sheet

Commercial name	Company	Dangerous components	Percent (%)	Groups
Twinky Star Lot #2107291	VOCO GmbH, Germany	BIS-GMA UDMA TEGDMA	10–25% 10–25% < 2.5%	Group 1: Berry; Group 2: Lemon; Group 3: Orange; Group 4: Green; Group 5: Gold; Group 6: Pink; Group 7: Blue; Group 8: Silver
Glasiosite Lot #2131116	VOCO GmbH, Germany	BIS-GMA TEGDMA BHT	Contains, quantity information is not available	Group 9: White-A2 shade
Ionolux Lot #2104097	VOCO GmbH, Germany	FLUORO ALUMINOSILICATE GLASS POLYACRYLIC ACID	50–100% 5–10%	Group 10: RMGIC
Arabesk Lot #2106783	VOCO GmbH, Germany	BIS-GMA UDMA TEGDMA	10–25% 5–10% 2.5–5%	Group 11: Micro-hybrid composite- Arabesk-A2 shade
GrandioSO Lot #2105341	VOCO GmbH, Germany	BIS-GMA TEGDMA BIS-EMA	2.5–5% 2.5–5% 2.5–5%	Group 12: Nano-hybrid composite-GrandioSO-A2 shade

Information on the composition(s) of resin restorative materials is included in the company's safety data sheet

The minimum sample size required to detect a significant difference was at least 11 in each group, considering type I error (alfa) of 0.05, power (1-beta) of 0.8, an effect size of 0.95, and two-sided alternative hypothesis (H1) [17]. A total of 156 samples, 13 samples in each group, were used for this study.

### Preparation of specimens

The specimens were prepared according to the instructions of the manufacturers. In order to prevent inter examiner variations, all study samples were prepared by a clinician (SA), who was trained and calibrated by an experienced clinician (GD). The standardized cylindrical specimens of the resin-based dental materials were prepared by placing the materials into a teflon mould with a diameter of 5 mm and 2 mm thickness. The resin materials were applied as a single increment of 2 mm. The surface was covered with a transparent plastic matrix strip (Kerr Hawe Stopstrip, Switzerland), pressed with 1 mm thick glass plate with finger pressure on the top height of the mold to extrude the excess material, and then the glass plate was discarded. The specimens were cured by using an LED light curing unit (CELALUX 2—VOCO, Cuxhaven, Germany) with a wavelength of 420–480 nm and a light intensity of 1300 mW/cm^2^ perpendicular to the surface in close contact [15]. According to the manufacturer’s instructions, compomer, RMGIC, and micro*-* and nano-hybrid composite specimens were cured for 40 s, 20 s, and 40 and 20 s, respectively. The consistency of the curing light intensity was verified using a radiometer (3 M ESPE Elipar™ S10) for each irradiation. The finishing and polishing procedures were performed with a 12-fluted carbide finishing bur (Hager &Meisinger GmbH, Neuss, Germany) and Sof-Lex polishing discs (3 M ESPE, St Paul, MN, USA), respectively. All specimens were rinsed with water for 10 s and then air-dried for 5 s.

### High-performance liquid chromatography

The 75% ethanol (Merck, HPLC grade) - 25% ultra pure water solution (HPLC Gradient Grade solvents) was prepared and stored at + 4 °C in the dark until the time of analysis.

Each specimen was immediately immersed in amber-colored glass vials containing 20 mL 75% ethanol/water solution and stored at 37 °C. HPLC–PDA measurements were carried out in every 1 h, 1 day, 7 days, 14 days, and 21 days after the immersion. After each interval, the resin discs were taken out from the solution, air-dried with the very mild stream of the air, and immersed in 20 mL of fresh ethanol/water solution. The solutions obtained after each interval were filtered through a 0.45 μm filter. An aliquot (20 μL) of the filtrate was used for injection into the HPLC–PDA system.

The determination of monomers was carried out using a Shimadzu HPLC (Shimadzu Technologies, Kyoto, Japan) equipped with photodiode array detector. Separations were performed using a Clipeus C18 5 µm reversed-phase column (250 mm × 4.6 mm). Isocratic elution was performed with 1 mL/min flow rate at 30ºC, and the injection volume was 20 μL. In this process, 80% acetonitrile (Merck, HPLC grade) / 20% ultra pure water mixture was used as a mobile phase. The dedector was set at 231 nm for all analytes because they exhibit significant absorption. 0.1, 1, 10, 50, and 100 µg/mL solutions of HEMA (Sigma-Aldrich, ≥ 99%), BIS-GMA (Sigma-Aldrich), TEGDMA (Sigma-Aldrich, ≥ 99%), and UDMA (Sigma-Aldrich, ≥ 97%) were prepared by appropriate dilution of aliquots of 1000 µg/mL to prepare the calibration curve. Additionally, limit of detection (LOD) and limit of quantification (LOQ) was calculated by multiplying 3.3 and 10 by the standard deviation of the blank solution(s) respectively (Table 2). Quantification was performed by comparing peak areas with those of monomer standards. The data observed were recorded as µg/mL (ppm). The calibration of the device, the extraction of the residual monomers from the resin samples, and their measurements were performed by an experienced examiner (YU).

**Table 2:** Analytical characteristics of monomer analyses by HPLC–PDA detection

	Regression equation	R^2^	Retention time (minute)	LOD (μg/mL)	LOQ (μg/mL)
HEMA	y = 2.9268x-0.003	0.9983	3.307	0.040	0.122
TEGDMA	y = 2.4733x-0.003	0.9999	4.124	0.050	0.151
UDMA	y = 5.2986x-0.003	0.9999	4.622	0.133	0.402
BIS-GMA	y = 3.1184x-0.004	0.9999	5.087	0.031	0.095

### Statistical analysis

The data were statistically analyzed using IBM SPSS Statistics for Windows, version 26.0. The results of the Shapiro–Wilk normality test showed that the data was normally distributed. One-way ANOVA with Bonferroni post hoc test was used to compare the amount of residual monomer among the groups. Repeated measures ANOVA with Bonferroni post hoc test was used to compare the amount of residual monomer among five time periods. Statistical analyses were carried out at a significance level of 0.05.

## Results

The mean and standard deviation values of the residual monomer released in µg/mL (ppm) are presented in Tables 3, 4, 5, and 6. There were statistically significant differences among the groups and among the different time periods in the same group in terms of the residual monomer release (*p* < 0.001). The residual monomer release continued until the 21st day. Of all the groups, the most released monomer was BIS-GMA.

**Table 3:** The mean and standard deviation values of the amount of HEMA released (μg/mL)

Group	1st hour	1st day	7th day	14th day	21st day	**p-value
1	0.021 ± 0.004^a^ (0.0002)	0.079 ± 0.005^b^ (0.0006)	0.150 ± 0.020^c^ (0.0012)	0.043 ± 0.006^d^ (0.0003)	0.029 ± 0.003^d^ (0.0002)	< 0.001
2	0.028 ± 0.005^ad^ (0.0002)	0.089 ± 0.011^b^ (0.0007)	0.152 ± 0.024^c^ (0.0012)	0.054 ± 0.010^a^ (0.0004)	0.035 ± 0.003^d^ (0.0003)	< 0.001
3	0.034 ± 0.003^ad^ (0.0003)	0.083 ± 0.008^b^ (0.0006)	0.146 ± 0.028^c^ (0.0011)	0.041 ± 0.007^a^ (0.0003)	0.033 ± 0.005^d^ (0.0003)	< 0.001
4	0.024 ± 0.002^a^ (0.0002)	0.061 ± 0.006^b^ (0.0005)	0.118 ± 0.007^c^ (0.0009)	0.031 ± 0.004^a^ (0.0002)	0.028 ± 0.005^a^ (0.0002)	< 0.001
5	0.081 ± 0.019^a^ (0.0006)	0.149 ± 0.024^b^ (0.0011)	0.203 ± 0.032^b^ (0.0016)	0.053 ± 0.006^a^ (0.0004)	0.026 ± 0.003^c^ (0.0002)	< 0.001
6	0.022 ± 0.005^a^ (0.0002)	0.060 ± 0.003^b^ (0.0005)	0.113 ± 0.011^c^ (0.0009)	0.033 ± 0.005^d^ (0.0003)	0.023 ± 0.003^a^ (0.0002)	< 0.001
7	0.017 ± 0.003^a^ (0.0001)	0.048 ± 0.004^b^ (0.0004)	0.091 ± 0.007^c^ (0.0007)	0.024 ± 0.004^a^ (0.0002)	0.014 ± 0.003^a^ (0.0001)	< 0.001
8	0.019 ± 0.001^a^ (0.0001)	0.055 ± 0.005^b^ (0.0004)	0.087 ± 0.004^c^ (0.0007)	0.025 ± 0.004^a^ (0.0002)	0.014 ± 0.0003^d^ (0.0001)	< 0.001
9	0.022 ± 0.003^ac^ (0.0002)	0.065 ± 0.014^b^ (0.0005)	0.102 ± 0.020^b^ (0.0008)	0.029 ± 0.003^a^ (0.0002)	0.022 ± 0.003^c^ (0.0002)	< 0.001
10	0.006 ± 0.001^a^ (0.0000)	0.025 ± 0.002^b^ (0.0002)	0.029 ± 0.005^b^ (0.0002)	0.009 ± 0.002^a^ (0.0001)	0.010 ± 0.003^a^ (0.0008)	< 0.001
11	0.025 ± 0.006^ad^ (0.0002)	0.071 ± 0.008^b^ (0.0005)	0.131 ± 0.020^c^ (0.0010)	0.038 ± 0.005^a^ (0.0003)	0.029 ± 0.003^d^ (0.0002)	< 0.001
12	0.016 ± 0.002^a^ (0.0001)	0.031 ± 0.004^bc^ (0.0002)	0.047 ± 0.011^c^ (0.0004)	0.017 ± 0.005^a^ (0.0001)	0.019 ± 0.006^ab^ (0.0001)	< 0.001
*p-value	< 0.001	< 0.001	< 0.001	< 0.001	< 0.001	

The numbers in parentheses are expressed as mmol/L

^*^One way ANOVA post Hoc bonferroni

^**^Repeated measures ANOVA post hoc bonferroni

^a,b,c,d^:Different letters indicate statistically *significant* difference at *p* < 0.05 in the same row

**Table 4:** The mean and standard deviation values of the amount of TEGDMA released (μg/mL)

Group	1st hour	1st day	7th day	14th day	21st day	**p-value
1	1.668 ± 0.183^ab^ (0.0058)	2.060 ± 0.175^a^ (0.0072)	1.487 ± 0.234^b^ (0.0052)	0.483 ± 0.052^c^ (0.0017)	0.387 ± 0.028^c^ (0.0014)	< 0.001
2	2.403 ± 0.562^ab^ (0.0084)	2.801 ± 0.519^a^ (0.0098)	1.507 ± 0.255^b^ (0.0053)	0.550 ± 0.061^c^ (0.0019)	0.362 ± 0.051^d^ (0.0013)	< 0.001
3	2.340 ± 0.439^ab^ (0.0082)	2.766 ± 0.366^a^ (0.0097)	1.499 ± 0.342^b^ (0.0052)	0.484 ± 0.056^c^ (0.0017)	0.404 ± 0.022^c^ (0.0014)	< 0.001
4	1.195 ± 0.222^ab^ (0.0042)	1.682 ± 0.220^a^ (0.0059)	1.052 ± 0.032^b^ (0.0037)	0.413 ± 0.021^c^ (0.0014)	0.341 ± 0.025^d^ (0.0012)	< 0.001
5	10.410 ± 1.880^a^ (0.0364)	6.391 ± 1.457^b^ (0.0223)	2.332 ± 0.352^c^ (0.0081)	0.543 ± 0.074^d^ (0.0019)	0.432 ± 0.021^d^ (0.0015)	< 0.001
6	1.341 ± 0.344^ab^ (0.0047)	1.779 ± 0.151^a^ (0.0062)	1.131 ± 0.125^b^ (0.0039)	0.388 ± 0.076^c^ (0.0014)	0.455 ± 0.034^c^ (0.0016)	< 0.001
7	0.776 ± 0.098^a^ (0.0027)	1.252 ± 0.100^b^ (0.0044)	0.852 ± 0.098^a^ (0.0030)	0.245 ± 0.024^c^ (0.0009)	0.392 ± 0.032^d^ (0.0014)	< 0.001
8	0.793 ± 0.068^a^ (0.0028)	1.446 ± 0.066^b^ (0.0051)	0.845 ± 0.048^a^ (0.0030)	0.275 ± 0.031^c^ (0.0010)	0.353 ± 0.018^d^ (0.0012)	< 0.001
9	1.169 ± 0.225^ab^ (0.0041)	1.725 ± 0.281^a^ (0.0060)	0.959 ± 0.189^b^ (0.0033)	0.286 ± 0.049^c^ (0.0010)	0.396 ± 0.033^d^ (0.0014)	< 0.001
10	0.000 ± 0.000^a^ (0.0000)	0.520 ± 0.064^b^ (0.0018)	1.131 ± 0.938^c^ (0.0039)	0.363 ± 0.056^d^ (0.0013)	0.303 ± 0.066^d^ (0.0011)	< 0.001
11	1.016 ± 0.115^a^ (0.0035)	1.502 ± 0.100^b^ (0.0052)	1.158 ± 0.149^a^ (0.0039)	0.405 ± 0.048^c^ (0.0014)	0.317 ± 0.029^d^ (0.0011)	< 0.001
12	1.520 ± 0.490^a^ (0.0053)	1.854 ± 0.621^a^ (0.0065)	1.056 ± 0.254^a^ (0.0037)	0.314 ± 0.056^b^ (0.0011)	0.000 ± 0.000^c^ (0.0000)	< 0.001
*p-value	< 0.001	< 0.001	< 0.001	< 0.001	< 0.001	

The numbers in parentheses are expressed as mmol/L

^*^One way ANOVA post hoc bonferroni

^**^Repeated measures ANOVA post hoc bonferroni

^a,b,c,d^:Different letters indicate statistically *significant* differences at *p* < 0.05 in the same row

**Table 5:** The mean and standard deviation values of the amount of UDMA released (μg/mL)

Group	1st hour	1st day	7th day	14th day	21st day	**p-value
1	5.012 ± 0.807^a^ (0.0107)	11.747 ± 0.970^b^ (0.0250)	7.883 ± 1.153^a^ (0.0168)	2.081 ± 0.356^c^ (0.0044)	1.852 ± 0.190^c^ (0.0039)	< 0.001
2	6.910 ± 1.445^ab^ (0.0147)	13.934 ± 2.208^a^ (0.0296)	9.112 ± 1.475^b^ (0.0194)	3.038 ± 0.454^c^ (0.0065)	2.302 ± 0.253^d^ (0.0049)	< 0.001
3	7.411 ± 1.445^ab^ (0.0157)	13.008 ± 1.869^a^ (0.0276)	8.806 ± 2.051^b^ (0.0187)	2.603 ± 0.676^c^ (0.0055)	2.194 ± 0.373^c^ (0.0047)	< 0.001
4	3.885 ± 0.331^a^ (0.0083)	8.947 ± 0.787^b^ (0.0190)	5.689 ± 0.417^c^ (0.0125)	1.568 ± 0.148^d^ (0.0033)	1.548 ± 0.190^d^ (0.0033)	< 0.001
5	27.371 ± 7.976^ab^ (0.0582)	28.431 ± 5.619^a^ (0.0604)	14.340 ± 2.335^b^ (0.0305)	3.283 ± 0.332^c^ (0.0070)	2.034 ± 0.186^d^ (0.0043)	< 0.001
6	4.213 ± 1.058^a^ (0.0090)	8.902 ± 1.058^b^ (0.0189)	5.895 ± 0.843^a^ (0.0125)	1.706 ± 0.393^c^ (0.0036)	1.606 ± 0.275^c^ (0.0034)	< 0.001
7	2.543 ± 0.730^a^ (0.0054)	6.320 ± 0.638^b^ (0.0134)	3.842 ± 0.538^c^ (0.0082)	0.975 ± 0.204^d^ (0.0021)	1.075 ± 0.160^d^ (0.0023)	< 0.001
8	2.735 ± 0.270^a^ (0.0058)	7.199 ± 0.501^b^ (0.0153)	4.290 ± 0.325^c^ (0.0091)	1.019 ± 0.119^d^ (0.0022)	0.972 ± 0.073^d^ (0.0021)	< 0.001
9	2.107 ± 0.493^a^ (0.0045)	4.936 ± 1.067^b^ (0.0105)	3.355 ± 0.781^ab^ (0.0071)	0.800 ± 0.144^c^ (0.0017)	0.834 ± 0.131^c^ (0.0018)	< 0.001
10	0.558 ± 0.117^ac^ (0.0045)	0.858 ± 0.215^a^ (0.0018)	1.116 ± 0.122^b^ (0.0024)	0.296 ± 0.037^c^ (0.0006)	0.000 ± 0.000^d^ (0.0000)	< 0.001
11	2.495 ± 0.544^a^ (0.0053)	5.806 ± 0.334^b^ (0.0123)	4.329 ± 0.526^b^ (0.0092)	1.101 ± 0.103^c^ (0.0023)	1.068 ± 0.073^c^ (0.0023)	< 0.001
12	0.232 ± 0.075^ac^ (0.0005)	0.355 ± 0.086^a^ (0.0008)	0.788 ± 0.142^b^ (0.0017)	0.204 ± 0.067^c^ (0.0004)	0.000 ± 0.000^d^ (0.000)	< 0.001
*p-value	< 0.001	< 0.001	< 0.001	< 0.001	< 0.001	

The numbers in parentheses are expressed as mmol/L

^*^One way ANOVA post hoc bonferroni

^**^Repeated measures ANOVA post hoc bonferroni

^a, b, c,d^:Different letters indicate statistically *significant* differences at *p* < 0.05 in the same row

**Table 6:** The mean and standard deviation values of the amount of BIS-GMA released (μg/mL)

Group	1st hour	1st day	7th day	14th day	21st day	**p-value
1	5.931 ± 0.686^a^ (0.0116)	14.792 ± 1.229^b^ (0.0289)	12.321 ± 1.641^b^ (0.0240)	3.155 ± 0.546^c^ (0.0062)	2.567 ± 0.322^c^ (0.0050)	< 0.001
2	7.881 ± 1.790^ac^ (0.0154)	17.206 ± 2.774^b^ (0.0336)	12.969 ± 1.975^a^ (0.0247)	4.242 ± 0.679^c^ (0.0083)	3.284 ± 0.399^d^ (0.0064)	< 0.001
3	8.465 ± 1.614^ab^ (0.0165)	15.876 ± 2.456^a^ (0.0310)	12.642 ± 2.683^b^ (0.0247)	3.750 ± 1.979^c^ (0.0073)	3.064 ± 0.554^c^ (0.0060)	< 0.001
4	4.237 ± 0.494^a^ (0.0083)	10.658 ± 1.023^b^ (0.0208)	8.587 ± 0.638^c^ (0.0168)	2.315 ± 0.188^d^ (0.0045)	2.163 ± 0.292^d^ (0.0042)	< 0.001
5	33.278 ± 10.622^ab^ (0.0649)	35.731 ± 7.434^a^ (0.0697)	20.557 ± 3.208^b^ (0.0401)	4.954 ± 0.519^c^ (0.0097)	3.021 ± 0.281^d^ (0.0059)	< 0.001
6	4.738 ± 1.335^a^ (0.0092)	10.750 ± 1.392^b^ (0.0210)	8.922 ± 1.218^c^ (0.0174)	2.526 ± 0.577^d^ (0.0049)	2.197 ± 0.411^e^ (0.0043)	< 0.001
7	2.784 ± 0.663^a^ (0.0054)	7.683 ± 0.907^b^ (0.0150)	5.999 ± 0.648^c^ (0.0117)	1.543 ± 0.257^d^ (0.0030)	1.413 ± 0.239^d^ (0.0028)	< 0.001
8	2.969 ± 0.306^a^ (0.0058)	8.586 ± 0.614^b^ (0.0168)	6.736 ± 0.666^c^ (0.0131)	1.592 ± 0.197^d^ (0.0031)	1.381 ± 0.142^e^ (0.0027)	< 0.001
9	3.310 ± 0.856^a^ (0.0065)	8.084 ± 1.840^b^ (0.0158)	6.587 ± 1.411^b^ (0.0129)	1.700 ± 0.292^a^ (0.0033)	1.472 ± 0.207^c^ (0.0029)	< 0.001
10	0.190 ± 0.035^a^ (0.0004)	0.745 ± 0.100^b^ (0.0015)	1.217 ± 0.105^c^ (0.0024)	0.315 ± 0.042^d^ (0.0006)	0.144 ± 0.025^a^ (0.0003)	< 0.001
11	5.287 ± 1.167^a^ (0.0103)	12.460 ± 0.996^b^ (0.0243)	10.587 ± 1.214^b^ (0.0207)	2.920 ± 0.252^a^ (0.0057)	2.450 ± 0.147^c^ (0.0048)	< 0.001
12	2.381 ± 0.781^ab^ (0.0046)	3.137 ± 1.167^a^ (0.0061)	2.894 ± 1.056^a^ (0.0056)	1.123 ± 0.368^b^ (0.0022)	0.801 ± 0.362^c^ (0.0016)	< 0.001
*p-value	< 0.001	< 0.001	< 0.001	< 0.001	< 0.001	

The numbers in parentheses are expressed as mmol/L

^*^One way ANOVA post hoc bonferroni

^**^Repeated measures anova post hoc bonferroni

^a, b, c, d^:Different letters indicate statistically *significant* differences at *p* < 0.05 in the same row

HEMA release showed the maximum increase on the 7th day in all the materials. The highest HEMA release was from the gold-colored compomer (0.203 ± 0.032 µg/mL on 7th day). HEMA release from RMGIC was less than the others in all time periods.

TEGDMA release showed the maximum increase at the end of the 1st hour or 1st day in colored compomers. The most TEGDMA release among the resin materials was gold-colored compomer (10.410 ± 1.880 µg/mL) at the end of the 1st hour, and it was statistically much higher than the others (*p* < 0.01). The green-colored compomer released more TEGDMA after the 1st hour, although it was not statistically significant, compared to the relese on the 1st day (*p* > 0.05). The other colored compomers, except the green and gold-colored compomers, released the highest TEGDMA on the 1st day. TEGDMA release from the RMGIC and the nanohybrid resin composite-GrandioSO could not be detected in the 1st hour and on the 21st day, respectively.

When UDMA release was evaluated, it was found that the gold-colored compomer was the material that showed the maximum UDMA release in all time periods, except the 21st day. The maximum UDMA release of all the materials, except the nanohybrid resin composite-GrandioSO was on the 1st day. For the nanohybrid resin composite-GrandioSO, the maximum UDMA release was on the 7th day. UDMA release from the RMGIC and the nanohybrid resin composite-GrandioSO could not be detected on the 21st day.

BIS-GMA monomer release showed the maximum increase on the 1st day in all the materials, except RMGIC. The material releasing the maximum BIS-GMA was the gold-colored compomer in all time periods, except the 21st day (*p* < 0.01). At the end of the 21st day, lemon, orange, and gold-colored compomer released more BIS-GMA monomer than the others.

**Fig. 1:**
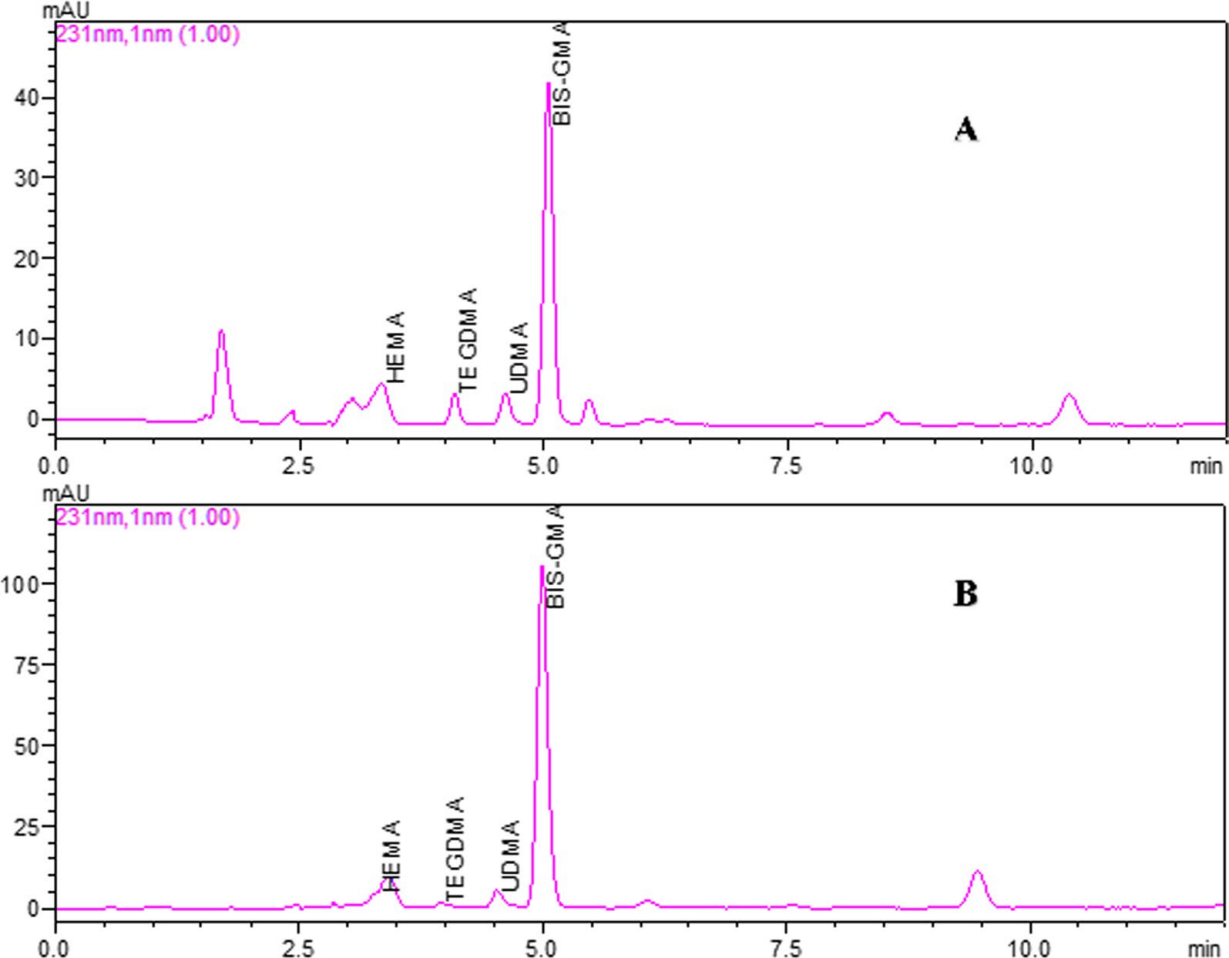
HPLC chromatograms of 10 µg/mL mix monomer standard solution (**A**) and sample (compomer 2, 7th day) (**B**)

HPLC chromatograms of 10 µg/mL mix monomer standard solution and sample are shown in Fig. 1.

## Discussion

In this in vitro study, the amount of BIS-GMA, TEGDMA, HEMA, and UDMA monomers released into 75% ethanol solution in the 1st hour and on the 1st, 7th, 14th, and 21st days after polymerization of compomers in different colors (a shade A_2_ compomer and 8 different color packable compomers), composites (shade A2) in different hybridization (nano-hybrid, micro-hybrid), and RMGIC, which are frequently used in pediatric dentistry, was investigated by using HPLC–PDA method.

It is recommended to irradiate the resin materials in layers of 2 mm to increase the polymerization efficiency [27]. In this study, 2 mm high teflon molds were used, taking into account the manufacturer's recommendations for the product use.

Some of the studies measuring the effectiveness of LCUs on the polymerization of resin based restorative materials indicated that Halogen LCUs were more effective [2, 25, 28, 29], while others stated that LED LCUs were more effective [18, 30]. In line with the manufacturer's recommendation, an LED LCU was preferred for polymerization in this study.

It is known that the oxygen inhibition layer formed on the surface of the restorative resin material after polymerization is rich in residual monomers [7]. Bezgin et al. [8] reported that Mylar strips did not prevent the formation of the oxygen inhibition layer, and finishing-polishing is still essential for the elimination of the resin-rich outer layer that can be the source of the unreacted monomers eluted to the oral cavity. Although transparent strips were used in our study, finishing and polishing processes were applied to the upper surfaces of the discs to remove the oxygen inhibition layer.

The most commonly used chromatographic methods for distinguishing the components released from resin-based materials are HPLC and Gas Chromatography / Mass Spectrometry (GC/MS) [19], and HPLC was preferred in this study.

It is known that saliva is the main factor in the dissolution of resin-based dental materials in the oral environment over time after application [31]. This dissolving effect of saliva in the oral environment is tried to be imitated with solvents such as acitonitrile, artificial saliva, water, ethanol and methanol in different proportions in vitro studies [31–33]. The US Food and Drug Administration (FDA) recommends a 75% ethanol-water solution, which is similar to the oral conditions, for the detection of residual monomers [34]. While artificial saliva barely penetrates the polymer network of the resin-based material [20, 21], ethanol has been used by many researchers because it penetrates the polymer network of the material, widening the gaps between the polymer chains and facilitating the release of unreacted monomers over time [8, 16, 20–22, 35]. Therefore, in this study, 75% ethanol—25% deionized water was used as the extraction medium to measure the release of the monomers.

The ethanol solution was not changed from the beginning to the end of the study in our previous study [26]. However, in this study, the solution was changed at the end of each time period based on the previous studies [20–22, 35]. Shahabi et al. [22] evaluated the effect of the volume (1 mL or 3 mL) and renewing of storage media (ethanol/water solution) on monomer (UDMA, BIS-GMA, TEGDMA) leachability from two dental composites using HPLC. They reported that saturation makes the storage media volume important factor in monomer elution and refreshing of storage media had significant effect on monomer release before the elution of 50% of total released monomer.

It has been reported that monomer release is high in resin based restorative materials at the beginning and this amount continues to decrease over time [21–23, 36]. The first mechanism of monomer elution is elution from the composite surface that occurs in the first 24 h. Subsequently monomer elution continues with a slower rate, since increasing the volume of polymeric chains and release of unreacted monomers from composite take substantial time [23]. There are studies examining the release of monomers from restorative resin materials in different time periods [21, 35, 37, 38]. Moreira et al. [38] indicated that the release of residual monomer continued until the 30th day. In the previous study conducted in our clinic, residual monomer release was evaluated at the end of 10 min, 1 h, 6 h, 1, 7, and 14 days [26]. Unlike our previous study, the 10th minute was eliminated from the evaluation periods and the final evaluation period was extended to 21 days. In this study, the measurement periods for the amount of residual monomer released from the resin-based materials were determined as 1 h, 1, 7, 14, and 21 days.

Although monomer elution from the resin-composites has been widely assessed [7, 15, 17–24], studies evaluating monomer elution from the compomers are very limited [8, 25, 26]. To the best of our knowledge, there are very few studies in the literature examining the amount of residual monomer released after polymerization of colored compomers and RMGIC. Botsali et al. [25] reported that there was a higher amount of HEMA release than TEGDMA release in their study with one RMGIC (Ketac N100) and two compomers (Dyract Extra and Twinkystar), and this release damaged fibroblast cells. Tunç et al. [28] stated that compomers are potentially toxic to human pulp fibroblasts and the type of curing unit will affect compomer toxicity. In our previous study, it was determined that BIS-GMA was the most released residual monomer, despite its high viscosity which makes it difficult to release in packable and flowable compomers, while TEGDMA was the least released monomer [26]. Residual monomer release continued on the 14th day and the compomer with the highest residual monomer release was the gold-colored compomer. It was concluded that color and viscosity affected the residual monomer release in compomers. Although that study was the first to examine the release of residual monomer according to the color of the compomers, it showed that not all the colors in the compomer color scale were examined as a limitation of the study [26]. In this study, which was carried out with all the colors in the compomer color scale in order to eliminate this limitation in the literature, it was concluded that color is important in residual monomer release. Dark colored compomers absorbing blue light are thought to have a greater depth of polymerization. Vandenbulcke et al. [3] reported that the polymerization depth of colored compomers could be affected by the amount and type of pigment. They found that the relatively darker shades (blue and green) had the greatest polymerization depths.

According to the previous studies, the toxicity for the following monomers was ranked as BIS‐GMA > UDMA > TEGDMA > HEMA (least toxic) [39, 40]. In this study, these four monomers were also investigated.

BIS-GMA was the most released monomer in all the groups except RMGIC. Ranasathien et al. [40] found the cytotoxic effect value of BIS-GMA as 9.35 μM/L (4.78 µg/mL) in their study on mouse fibroblasts. In a study, exposure of dental pulp cells to BIS-GMA at concentrations of 0.075 mmol/L markedly affected the viable cell number with 40% of inhibition [39], while in another study, it was reported that BIS-GMA at concentration of 0.087 mmol/L causes 50% reduction (half maximal effect concentration: EC50) of cell viability on human gingival fibroblasts [14]. In this study, BIS-GMA concentration in gold-colored compomer (Twinky Star) – in the 24th hour, which was the highest concentration with 35.731 µg/mL (0.0697 mmol/L), was found to be either lower or greater than the toxic concentrations obtained in some previous studies [14, 39, 40].

HEMA release showed a maximum increase on the 7th day in all the groups. Altıntaş and Üşümez, [41] investigated the residual monomer release from resin cements, and reported the HEMA release amount from Nexus 2 (Kerr/Italy) cement to be 117 µg/mL in the 10th minute, and 440 µg/mL on the 21st day. In the same study, they were measured as 98.15 µg/mL in the 10th minute and 142.61 µg/mL on the 21st day for Rely X Arc (3 M ESPE/Germany). Botsali et al. [25] found HEMA release from RMGIC (Ketac N100) to be 7.1 µm/L in the 4th hour and 16.8 µm/L in the 24th hour. On the other hand, in our study, the amount of HEMA released from the resin cement of Ionolux (VOCO, Germany) was found to be 0.006 µg/mL in the 1st hour, 0.025 µg/mL (0.0002 mmol/L) on the 1st day, and 0.010 µg/mL (0.0008 mmol/L) on the 21st day. HEMA release from RMGIC is less than other materials in all time periods. This situation may be due to the interaction of HEMA molecules with water, considering that HEMA is highly hydrophilic and the solution consists of 75% ethanol- 25% water [41]. Although HEMA is listed by the manufacturers as a component of RMGIC, it is not listed as a component of compomer. However, Geurtsen et al. [16], Bezgin et al. [8], and Botsali et al. [25] confirmed its presence in compomers. In this study, HEMA release was determined from compomers, and the highest HEMA release was from the gold-colored compomer. (0.203 ± 0.032 µg/mL–0.0016 mmol on the 7th day). Bezgin et al. [8] explained the presence of HEMA in the compomer by stating that manufacturers may keep the components with concentrations lower than 1% in their products confidential as it is a trade secret, and also ingredients in the Material Safety Data Sheet (MSDS) are sometimes insufficient. However, HEMA release could be a degradation product from UDMA, which is an ingredient in restorative materials [8, 16, 24]. Toxic concentration 50 (TC50) of HEMA ranged from 3.6 to 11.2 mmol/L with different cell lines in various studies [42–44]. In this study, no material reached the toxic concentrations obtained for HEMA in some previous studies [42–44].

TEGDMA is a low molecular weight monomer used to reduce the viscosity of BIS-GMA and UDMA. Sonkaya et al. [17] reported that the use of TEGDMA (co)monomer in dental composites reduced the monomer release. Of the resin-based dental materials used in this study, compomers contain less than 2.5% and composites contain between 2.5–5% TEGDMA. In our previous study, the most TEGDMA-releasing material among the packable compomers was the gold-colored compomer, and A_2_ shade compomer was found to be statistically much higher than the blue and pink-colored packable compomers [26]. In this study, the gold-colored compomer was the material that released the most TEGDMA with 10.410 µg/mL (0.0364 mmol/L) at the end of the 1st hour. TEGDMA release from RMGIC and nanohybrid resin composite-GrandioSO could not be detected at the end of 1 h and 21 days, respectively. Reichl et al. [45] reported the EC50 values decreased from about 5 mmol/L (6 h) to about 0.6 mmol/L (48 h) for HEMA and from about 3 mmol/L (6 h) to about 0.4 mmol/L (48 h) for TEGDMA in their cytotoxicity study. The effective dose that reduced the number of cell viability to 50% for TEGDMA was reported to be 0.26 mmol/L on human pulp fibroblasts [39] and 3.46 mmol/L on human gingival fibroblasts [14]. The TEGDMA concentrations of all the materials were found to be lower than the toxic concentrations obtained for TEGDMA in some previous studies [14, 39].

Although its molecular weight is close to that of BIS-GMA (512 g/mol), UDMA (470 g/mol) is highly viscous. In addition, despite having the same proportion as BIS-GMA in compomers, UDMA release is much lower than BIS-GMA. This is associated with its viscous structure. Reichl et al. [14] reported that UDMA at concentration of 0.106 mmol/L causes 50% reduction of cell viability (EC50) on human gingival fibroblasts. In this study, the highest UDMA release was observed on the 1st day in gold-colored compomers with 0.060 mmol/L.

Nanotechnology is the creation of macroscale structures by various processes of materials. This brings a more homogeneous matrix distribution with smaller particles and reduces the monomer matrix volume. As a result, the negative properties of the composite such as residual monomer release and polymerization shrinkage are reduced [17]. In general, residual monomer release was greater in microhybrid composites than in nanohybrid composites in this study. For TEGDMA, the situation was opposite in the first hour and on the first day. This difference in the residual monomer release taking place between micro-hybrid and nano-hybrid composites is thought to be due to the differences in filler particle type and monomer ratios specified by the manufacturer. De Angelis et al. [15] measured eluted monomer from GrandioSO (VOCO) nanohybrid composite after one day and 14 days using HPLC. They reported that the observable levels of TEGDMA were found only after 24 h (7.9 ng/mL), while the levels of BIS-GMA were about 4500 ng/mL after 24 h and 3500 ng/mL after 14 days. In this study, the levels of TEGDMA released from GrandioSO composite were 1.854 and 0.314 μg/mL after 24 h and 14 days, respectively, and it could not detected on the 21st day. Morever, the levels of BIS-GMA released were 3.137 and 1.123 μg/mL after 24 h and 14 days, respectively.

This study, in which all existing colors of resin based colored compomers were evaluated in terms of residual monomer release, will shed light on future studies. The limitation of the previous study on compomers [26], in which a limited number of colors were included, was resolved in this study. However, only one commercial company's filling materials and LED LCU were used in this study (VOCO®). The studies using the products of different companies should also be included. However, the strength of the study was the evaluation of compomers in all colors, composites with different hybridization properties, and RMGIC commonly used in pediatric dentistry in terms of residual monomer release by HPLC for the first time. In addition, the fact that it was conducted in vitro is another limitation of the study, and long-term clinical studies to be performed in saliva and gingival crevicular fluid will add valuable information to the literature.

## Conclusion

Although the factors affecting polymerization were standardized and the manufacturer's recommendations were followed, there were significant differences in residual monomer release between resin-based filling materials. Since the material that releases the most residual monomer is the gold-colored compomer, pedodontists should prefer it the least as a restorative material in children, especially in deep cavities or cavities close to the gingiva. Since RMGIC releases less residual monomer, its use can be expanded in pedodontic clinics in appropriate cases. Further studies are needed on the cytotoxic effects of restorative materials due to residual monomer release.

## Data Availability

The data that support the findings of this study are available from the data archive of the laboratory where the research was conducted, but restrictions apply to the availability of these data, which were used under license for the current study, and so are not publicly available. Data are however available from the coauthor Yılmaz Uğur upon reasonable request and with permission of the Apricot Research Institute, Malatya, Turkey.

## Abbreviations

RMGIC: Resin modified glass ionomer cements
HEMA: Hydroxy ethyl methacrylate
BIS-GMA: Bisphenol-A glycidyl methacrylate
TEGDMA: Tri ethylene glycol dimethacrylate
UDMA: Urethane dimethacrylate
BPA: Bisphenol-A
FDA: Food and drug administration
LED: Light emitting diodes
LCU: Light-curing units
